# Recovery Effects of a 180 mT Static Magnetic Field on Bone Mineral Density of Osteoporotic Lumbar Vertebrae in Ovariectomized Rats

**DOI:** 10.1155/2011/620984

**Published:** 2010-09-28

**Authors:** Shenzhi Xu, Hideyuki Okano, Naohide Tomita, Yoshito Ikada

**Affiliations:** ^1^Department of Mechanical Engineering and Science, Graduate School of Engineering, Kyoto University, Kyoto 606-8502, Japan; ^2^Department of Sciences, PIP Co., Ltd., Tokyo 101-8528, Japan; ^3^Research Center for Frontier Medical Engineering, Chiba University, Chiba 263-8522, Japan; ^4^Department of Indoor Environmental Medicine, Nara Medical University, Nara 634-8521, Japan

## Abstract

The effects of a moderate-intensity static magnetic field (SMF) on osteoporosis of the lumbar vertebrae were studied in ovariectomized rats. A small disc magnet (maximum magnetic flux density 180 mT) was implanted to the right side of spinous process of the third lumbar vertebra. Female rats in the growth stage (10 weeks old) were randomly divided into 4 groups: (i) ovariectomized and implanted with a disc magnet (SMF); (ii) ovariectomized and implanted with a nonmagnetized disc (sham); (iii) ovariectomized alone (OVX) and (vi) intact, nonoperated cage control (CTL). The blood serum 17-*β*-estradiol (E_2_) concentrations were measured by radioimmunoassay, and the bone mineral density (BMD) values of the femurs and the lumbar vertebrae were assessed by dual energy X-ray absorptiometry. The E_2_ concentrations were statistically significantly lower for all three operated groups than those of the CTL group at the 6th week. Although there was no statistical significant difference in the E_2_ concentrations between the SMF-exposed and sham-exposed groups, the BMD values of the lumbar vertebrae proximal to the SMF-exposed area statistically significantly increased in the SMF-exposed group than in the sham-exposed group. These results suggest that the SMF increased the BMD values of osteoporotic lumbar vertebrae in the ovariectomized rats.

## 1. Introduction

Yasuda et al. [1, 2] reported that the electrical current accelerated callus formation while Bassett et al. [3] showed that 34 patients with infantile nonunions associated with congenital “pseudoarthrosis” completed the clinical treatment with pulsed electromagnetic fields (PEMFs). An analysis of the results revealed that 17/34 (50%) patients achieved complete healing with biomechanically sound union, while failure was noticed in 10/34 patients (29%). These results imply that the exposure to PEMF induces an increase in bone formation [3–5]. It appears that a certain relationship exists between the PEMF and the electric current [6, 7]. Yonemori et al. [6] reported that the alkaline phosphatase and proliferative activities of osteoblast were significantly higher both in the direct current- (DC-) stimulated group and in the PEMF-stimulated group, when a Kirshner wire was inserted at 14 days after surgery than in three other groups, that is, the PEMF alone, the Kirshner wire insertion alone, and the intramedullary drilling.

The effects of static magnetic fields (SMFs) on bone metabolism have been reported [8–11], and these effects are known to be different from those of PEMF through different mechanisms. PEMF may generate an electric current in the tissue to stimulate some biological cascades, while SMF creates no detectable electrical potential in blood flow and hemodynamics at field levels <5 T [12, 13]. Because the effects of SMF are not dependent on electric energy, there are no heat and electric hazards on tissues [8]. This makes SMF a potential orthopaedic tool for long-term local exposure [9]. In Japan, in accordance with the guidelines of Ministry of Health, Labour and Welfare (Notification no. 119/1998), the magnetic flux densities produced by magnets ranging 35 mT to 200 mT have been accepted as therapeutic modalities. In particular, the magnetic flux densities regarding clinical applications have been gaining popularity are often referred to as ranging from 130 mT to 190 mT. Therefore, we have selected a 180 mT magnetic flux density (peak value) in a series of our studies on potential orthopaedic applications [9–11], including the present study. Our experimental studies with exposure to the gradient 180 mT SMF have demonstrated that the decrease in BMD values caused by ischemia induced upon operative invasion or artery ligation could be recovered by implantation of a magnetized rod accompanying SMF, together with an increase in mechanical strength of bone [9–11]. When collateral circulation was evaluated by injection of microspheres into the abdominal aorta at the 3rd week after ligation, the bone implanted with a magnetized rod showed a larger amount of trapped microspheres than that with a nonmagnetized rod [11]. This tendency was similar to that of the BMD values in the SMF-exposed ischemic bone. These studies support the assumption that the local BMD values near to a magnet was increased by the gradient 180 mT SMF. The previous studies also revealed that the gradient 180 mT SMF increased the BMD values by promoting the recovery of small-sized blood vessels with profound formation of collateral circulation [10, 11]. As a series of studies on the bone formation by SMF exposure, the present study attempted to investigate the effect of SMF on the recovery of osteoporosis using an ovariectomized animal model.

## 2. Methods

Ten-week-old female Wistar rats weighing 229.7 ± 16.9 g were used for this study (Charles River Laboratories Japan Inc., Kyoto, Japan). All animal experiments were approved by the Kyoto University Animal Research Committee, and all the experimental procedures were conducted in accordance with the guiding principles of the *Guide for the Care and Use of Laboratory Animals* published by the National Institutes of Health (NIH Publication.85-23).

**Figure 1:**
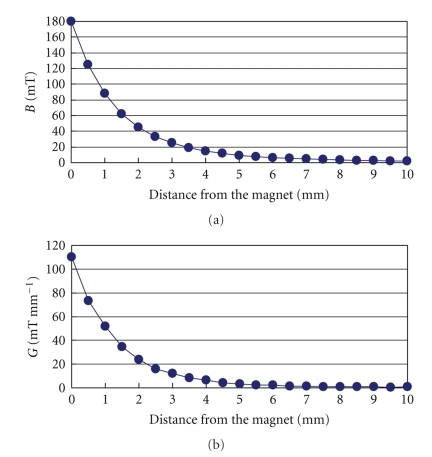
(a) Spatial distribution of the magnetic flux density values. (b) Spatial distribution of the magnetic gradient values.

The implanted magnets were made of SmFeN magnetic materials by kneading of SmFeN magnetic powders with polyamide as the binder, and then by injection molding the kneaded product (New Tech Co., Ltd., Nagano, Japan). The surface of all the magnets was homogeneously coated with polytetrafluoroethylene. Every piece of magnets had a diameter of 5.2 mm, a thickness of 2.5 mm, a weight of 165 mg, a maximum magnetic flux density (*B*_max _) of 180 mT, and a maximum magnetic gradient (*G*_max _) of 110 mT mm^−1^. The magnetic flux density was measured from the magnet using a gaussmeter (Model 4048, Hall probe A-4048-002, Bell Technologies) (Figure 1(a)). The magnetic gradient was calculated as described elsewhere [14] (Figure 1(b)).

**Figure 2:**
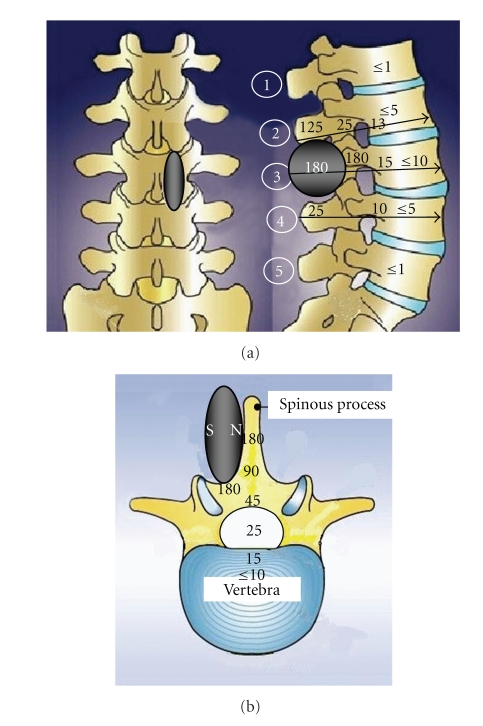
A magnet was implanted to the right side of spinous process of the third lumbar vertebra (L_3_). (a) Spatial distribution of the magnetic flux density values in lumbar vertebrae (unit: mT). Arrows show the direction of DXA beam under measurement after removal of the magnet. Encircled number indicates each lumbar vertebra. (b) Spatial distribution of the magnetic flux density values in a lumbar vertebra L_3_ (unit: mT). N: north-seeking side; S: south-seeking side.

After general intraperitoneal (i.p.) anesthesia with medetomidine (180 mg kg^−1^) and midazolam (1.25 mg kg^−1^), bilateral ovaries were extracted and a disc magnet was surgically implanted to the right side of spinous process of the third lumbar vertebra (L_3_) (Figure 2). Spatial distribution of the magnetic flux density values in L_3_ (whole lumbar vertebra including spinous process) was 180 mT or less (*B*_max_ = 180 mT). The *B*_max_ values in the second lumbar vertebra (L_2_) and the fourth lumbar vertebra (L_4_) were 125 mT and 25 mT, respectively. The north-seeking side of the magnet was directed to the right side of spinous process of L_3_. The magnet was fixed by surgical suture and medical grade glue. The sham magnet without magnetization was also prepared for implant use with the same size and materials as those of the magnet. The sham magnet was implanted in the same position of another rat to compare the effect with the magnet.

All rats were randomly assigned to one of four groups: (i) ovariectomized and implanted with a disc magnet (SMF); (ii) ovariectomized and implanted with a nonmagnetized disc (sham); (iii) ovariectomized alone (OVX); (vi) intact, nonoperated cage control (CTL). 

After the operation, two rats were housed together in one cage (LWH, 340 × 240 × 170 mm) using the same method as described in our previous report [13] and free access to water and standard pellet food was allowed. All free-moving the animals were bred at 25 ± 1°C  and 55 ± 5% relative humidity for 6 weeks. At the 6th week after the implantation, exsanguination was carried out from the abdominal aorta under general i.p. anesthesia, and then the femurs and lumbar vertebrae were taken out from the second (L_2_) to the fourth (L_4_).

Measurement items include (i) concentrations of blood serum 17-*β*-estradiol (E_2_, radioimmunoassay (RIA), FALCO Bio System, Kyoto, Japan) and (ii) BMD values of the femurs and lumbar vertebrae (each whole vertebra including the spinous process) (dual energy X-ray absorptiometry (DXA), Aloka DCS-600. SYS-D 162-V 6.0. Aloka, Tokyo, Japan). The BMD values were measured after removal of the magnet.

Statistical analysis was performed using the Kruskal-Wallis test and the Mann-Whitney *U* test (StatView 5.0, SAS Institute Inc., USA) for each of the measurements (*P* < .05). All of the data were expressed as the mean ± S. D.

## 3. Results

**Figure 3:**
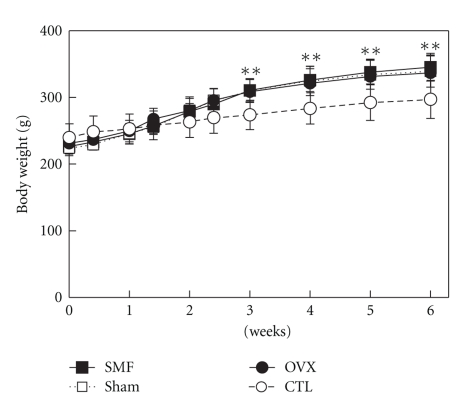
The rat body weight changes after ovariectomy or nonovariectomy. SMF: ovariectomized and implanted with a disc magnet; Sham: ovariectomized and implanted with a nonmagnetized disc; OVX: ovariectomized alone; CTL: intact (nonoperated) cage control. *n* = 10 in each group. **P < .01 versus CTL.

The body weights of all three operated groups were significantly higher from the 3rd week than that of the CTL group without operation (*P* < .01, Figure 3). The ovariectomy-increased body weight (obesity) is associated with estrogen deficiency [15]. Therefore, the operation was successful and the animal model for osteoporosis can be used for testing the SMF effect on the recovery of osteoporosis. 

**Figure 4:**
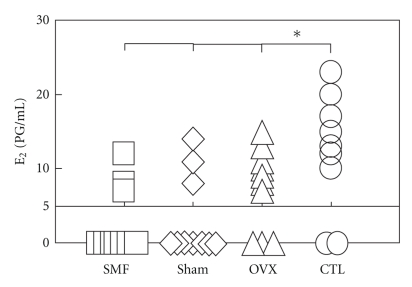
The E_2_ concentrations in the rat blood serum 6 weeks after ovariectomy or nonovariectomy. SMF: ovariectomized and implanted with a disc magnet; Sham: ovariectomized and implanted with a nonmagnetized disc; OVX: ovariectomized alone; CTL: intact (nonoperated) cage control. *n* = 10 in each group. *P < .05 versus CTL. no. of rats under the detection limit (<5 pg/mL): SMF, *n* = 7; Sham, *n* = 7; OVX, *n* = 3; CTL, *n* = 2. These E_2_ levels were shown as “0” at the E_2_ concentration.

The blood serum E_2_ concentrations of all three operated groups were significantly lower at the 6th week than those of the CTL group (*P* < .05, Figure 4). However, no significant difference was observed in the E_2_ concentration between the SMF-exposed and sham-exposed groups.

**Figure 5:**
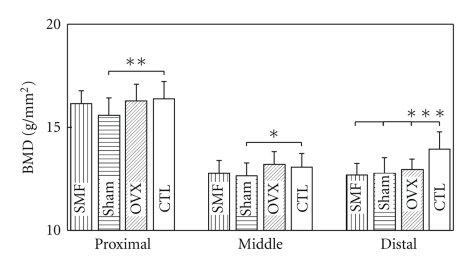
The BMD values of the rat femurs 6 weeks after ovariectomy or nonovariectomy. SMF: ovariectomized and implanted with a disc magnet; Sham: ovariectomized and implanted with a nonmagnetized disc; OVX: ovariectomized alone; CTL: intact (nonoperated) cage control. *n* = 20 in each group. *P < .05; **P < .01; ***P < .001 versus CTL.

The BMD values of the femurs at the distal region of all three operated groups were significantly lower than those of the CTL group (*P* < .001, Figure 5). However, no significant difference in the BMD values was noted for any part of the rat femurs between the SMF-exposed and sham-exposed groups.

**Figure 6:**
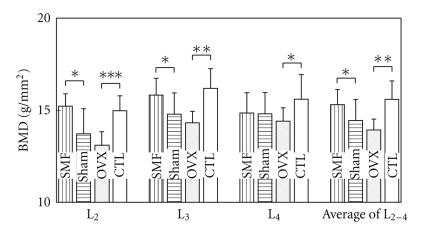
The BMD values of the rat lumbar vertebrae L_2_, L_3_, and L_4_ 6 weeks after ovariectomy or nonovariectomy. SMF: ovariectomized and implanted with a disc magnet; Sham: ovariectomized and implanted with a nonmagnetized disc; OVX: ovariectomized alone; CTL: intact (nonoperated) cage control. *n* = 10 in each group. *P < .05; **P < .01; ***P < .001 versus sham or CTL.

The BMD values of the second and the third lumbar vertebrae (L_2_, L_3_), and the total average BMD values from the second to the fourth lumbar vertebrae (L_2-4_) of the OVX group were significantly lower than those of the CTL group (L_2_, *P* < .001; L_3_, *P* < .01; L_4_, *P* < .05; L_2-4_, *P* < .01, Figure 6). The BMD values of the same vertebrae were significantly higher in the SMF group than those in the sham group (L_2_, *P* < .05; L_3_, *P* < .05; L_2-4_, *P* < .05, Figure 6). The BMD values of the SMF and CTL groups exhibited approximately the same level, when SMF exposure continued for 6 weeks.

## 4. Discussion

In contrast to our previous studies in which a rod type magnet was implanted into the rat femur by perforation using the magnet itself [10, 11], the present study attempted to implant a disc type magnet to the osseous surface of the third lumbar vertebra of ovariectomized rats with an aim to get more clinical relevant information. As matured rats are reported to be stable at the level of estrogen in an ovariectomized animal model [16, 17], relatively young rats (10-week old, approximately 230 g) in the growth stage were used in this study to examine the SMF effects on the concentration change of estrogen.

The blood serum E_2_ concentrations and BMD values were significantly lowered after ovariectomy compared with those of the CTL group (OVX versus CTL; Figures 4–6) and the body weights were significantly higher than those of the CTL group (Figure 3). These operation results suggest that this osteoporotic animal model can be used to evaluate the treatment of osteoporosis. When the E_2_ concentrations were under 5 pg/ml, those levels could not be detected with our RIA method due to the detection limit of sensitivity. The numbers of rats under the detection limit were found to be 7 in the SMF group, 7 in the sham group, 3 in the OVX group, and 2 in the CTL group, and these E_2_ concentrations were all shown as “0” (Figure 4). Turner et al. [18] reported that when E_2_ was administered in the form of a subcutaneous implant in ovariectomized ewes, the BMD values were significantly higher at the 5th lumbar vertebra (L_5_), calcaneus and distal radius at 12 months. Concerning the E_2_ levels in our study, however, no significant recovery after a 6-week exposure to SMF was observed. 

The BMD values of the second and third lumbar vertebrae (L_2_, *B*_max_ = 125 mT, *G*_max_ = 73  mT mm^−1^; L_3_, *B*_max_ = 180 mT, *G*_max_ = 110 mT mm^−1^) were significantly higher in the SMF group than those in the sham group, whereas there was no significant difference in those of fourth lumbar vertebra (L_4_, *B*_max_ = 25 mT, *G*_max_ = 12 mT mm^−1^) between the SMF-exposed and sham-exposed groups (Figure 6). This finding suggests that exposure of lumbar vertebrae to relatively stronger and gradient SMF (*B*_max_ ≥ 125 mT, *G*_max_ ≥ 73 mT mm^−1^) increased the BMD values of the lumbar vertebrae proximal to the SMF-exposed area without any significant influence on the E_2_ levels. These results are consistent with some theories of magnetic gradient: even though in the moderate-intensity range, gradient SMF has been shown to have significant biological effects [19–21]. 

The BMD values can also increase with gravitational acceleration. For instance, Rubin et al. [22] reported that after mechanically stimulating the hindlimbs of adult sheep on a daily basis for a year with 20-minutes bursts of very-low-magnitude, high-frequency vibration (the peak-to-peak amplitude of the strain generated was about 5 *μ*strain, 30 Hz), the density of the spongy (trabecular) bone in the proximal femur was increased significantly by 34.2% compared with the CTL group. Because the experimental period was relatively long as a year, the effect might become so evident. With regard to comparatively short temporal experiments on mechanical stimulation, Kohles et al. [23] continuously centrifuged rats for 14 days at twice gravitational acceleration (2 g) on a 12.75 foot radius centrifuge. They found significant increases not only in the Young's moduli and shear moduli, but also in the ratio of transverse to axial strain (Poisson's ratio) [23].

The rat stands straight often in a small cage. The lumbar vertebrae and femurs are both weight-bearing bones, with a lower mechanical load on the lumbar vertebrae than on the femurs. In the present study, the body weights were significantly higher (13%) for all three operated groups than for the nonoperated CTL group (Figure 3). The femur is the longest bone of the rat body and its main biomechanical function is to bear the body weight and move the body. We observed no significant difference in the BMD values of the femurs among the three operated groups, whereas at the distal region the BMD values in the three operated groups were significantly lower than that in the nonoperated CTL group (Figure 5). The comparison of the experimental condition of Rubin et al. (2001) [22] with that of the present study implies that the increased weight gain may not be related to the increased BMD values of the femurs. 

Only the BMD values of the lumbar vertebrae proximal to the SMF-exposed area were significantly higher in the SMF group than those in the sham group, which is approximately the same level as the nonoperated CTL group (Figure 6). These results suggest that the SMF would induce local bone formation to prevent bone degradation.

Using the same SMF intensity as our study, Nagai et al. [24] exposed an SMF at *B*_max_ of 180 mT to young rats (4 weeks old) for 7–21 days and found that the ectopic bone formation induced by purified bone morphogenetic protein (BMP) was promoted, compared with old rats (18 months old). They suggested that the mechanism of SMF effect might be related to an increase in blood vessel density in bone. Our previous study evaluated subchronic effects of local application of an SMF at *B*_max_ of 180 mT to the cutaneous microcirculation within a rabbit ear chamber under a conscious condition [25]. During an experimental period ranging from 24 hours to 4 weeks, SMF exposure for 1–3 weeks significantly induced long-lasting vasodilation with enhanced vasomotion as compared with the sham exposure [25]. Acute exposure to SMF in the field levels higher than 1 mT affected the muscle microcirculation by significantly increasing the peak blood velocity [26]. 

To our knowledge, however, as regards the effects of SMF on increasing BMD, there have been no other publications except for our own. Puricelli et al. [27, 28] carried out the histological analysis of the SMF-exposed region of bone graft in rats and showed that the SMF at *B*_max_ of 4 mT for at least 15 days healing process and stimulated bone neoformation. Moreover, Aydin and Bezer [8] examined the SMF effects on the osteotomized rabbit femur by histological analysis and BMD testing. Their study confirmed that an intramedullary implant with an SMF at *B*_max_ of 24 ± 2 mT improved bone healing in the first two weeks radiologically and that the configuration difference in magnetic poles had an effect on the bone healing process [8]. However, there was no significant SMF effect on BMD values even at the 4th week [8]. Bekhite et al. [29] found that in mouse fetuses, exposure of pregnant mice to a uniform SMF of 1 mT for 8 hours per day from the 3rd day of gestation till day 20 increased BMD but SMF of 10 mT decreased BMD, which was abolished in the presence of a free radical scavenger, Trolox. They suggest that SMF could modulate BMD via a reactive oxygen species- (ROS-) dependent upregulation of vascular endothelial growth factor (VEGF) expression [29].

As regards the SMF effects on hemodynamic function, increased knowledge (except for our own publications) may have significant therapeutic potential [30–37] and possible health effects [38]. For example, SMF therapy using moderate-intensity SMF (1 mT–1 T) could be useful for circulatory diseases, including ischemic pain, inflammation, and hypertension, primarily due to the modulation of blood flow and/or blood pressure through the nervous system. Regarding the relationship between the hemodynamics and nervous system, it is well known that the vasoconstriction is mediated mainly through sympathetic activity and adrenergic pathways. In contrast, the vasodilation is mediated mainly through parasympathetic activity and cholinergic pathways and, more specifically, the acetylcholine- (ACh-) induced vasodilation is induced by endothelium-derived nitric oxide (NO).

**Figure 7:**
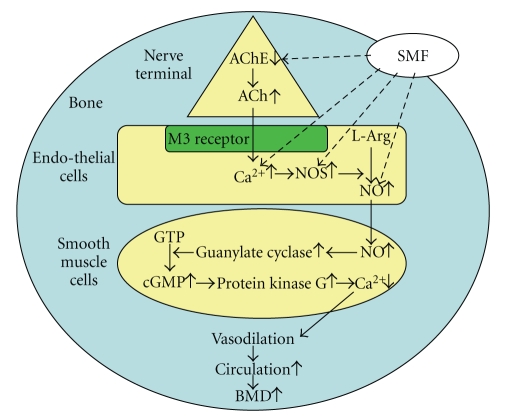
A hypothetical relationship diagram between SMF and hemodynamics that explains the possible mechanisms. Abbreviations: AChE: acetylcholinesterase; Ach: acetylcholine; NOS: nitric oxide synthase; NO: nitric oxide.

Li et al. [36] reported significant enhancement of the endothelial-related metabolic activity (0.01–0.05 Hz) in the skin stressed by pressure loading over the trochanter area upon exposure to an SMF at *B*_max_ of 30 mT. The modulating effect of SMF on the skin blood flow hemodynamics might be related to the vascular tone modified by prolonged compressive loading [36]. Takeshige and Sato [37] suggested a mechanism of SMF action for the promotion of blood flow hemodynamics that an SMF at *B*_max_ of 130 mT might inhibit acetylcholinesterase (AChE). Recovery of circulation is assumed to be partly due to the enhanced release of acetylcholine (ACh) by the SMF exposure, activating the cholinergic vasodilator nerve endings innervated to the muscle artery [37]. The inhibitory effect of SMF on AChE was also observed in the magnetic flux density of 0.8 mT or more [39]. In addition, it is also suggested that an SMF at *B*_max_ of 5.5 mT should have a potential to counteract the action of a nitric oxide synthase (NOS) inhibitor L-NAME, presumably via increased endogenous ACh release [40, 41]. The increased (upregulated) effect of a 120 *μ*T SMF on endothelial nitric oxide synthase (eNOS) expression was also confirmed in human umbilical vein endothelial cells (HUVEC) [42]. To achieve increased blood flow and circulation in bone and bone marrow, and thereby to further improve BMD, a hypothetical relationship diagram between SMF and hemodynamics that explains the possible mechanisms is shown in Figure 7. It is possible that improved bone blood circulation caused by SMF exposure may result in improved blood supply with bone growth factors such as BMP, to the osteoporotic lumbar vertebrae in the vicinity of the magnet, leading to an improvement of BMD values.

In vitro studies using bone cell culture systems may have some contribution to identification of biochemical factors in bone metabolism, as the SMF of moderate intensity (mT range) could induce osteoblastic differentiation at an early stage [43–52]. Kim et al. [43] showed that SMF as low as 10 mT affected cell attachment and proliferation in human osteosarcoma TE-85 cells. With regard to the mechanisms of SMF action for increased bone formation, Yuge et al. [44] and Yamamoto et al. [45] suggested that moderate-intensity SMF (30, 50 and 160 mT) affected the dynamics of intercellular calcium flowing into the cytoplasm of human and rat osteoblast. Shimizu et al. [46] reported that moderate-intensity SMF (30 and 80 mT) increased bone sialoprotein (BSP) transcription through a tyrosine kinase-dependent pathway in rat osteoblast-like cells, and that the SMF effects were mediated through a juxtaposed fibroblast growth factor-2 response element, and a pituitary-specific transcription factor-1 motif in the proximal promoter of the BSP gene. Huang et al. [47] further suggested that exposure to an SMF at *B*_max_ of 400 mT for 12–72 hours affected osteoblastic maturation by up-regulating early local factors, such as transforming growth factor (TGF)-*β*1, type I collagen, osteopontin, and alkaline phosphatase. Yang et al. [48] suggested that a uniform SMF of 0.4 T could impinge on osteoblastic differentiation via a Ca^2+^/calmodulin-dependent mechanotransduction pathway.

Other reports indicate that an SMF of strong intensity higher than a few Tesla has bioeffects [8, 9, 53–62]. For instance, an SMF of strong intensity with an extremely high magnetic gradient (8 T, 400 T^2^/m) could induce some bioeffects on paramagnetic hemoglobin by magnetic attraction in a high gradient or diamagnetic hemoglobin by magnetic repulsion in a high gradient, retarding the mean blood velocity in peripheral circulation, partly due to the asymmetric distribution of erythrocytes with different magnetic susceptibilities, and magnetically induced movement of diamagnetic water vapor at the skin surface, which may lead to a skin temperature decrease [58]. Because our applied SMF at *B*_max_ of 180 mT was much lower in the magnetic force compared with the SMF of several Tesla, different mechanisms might exist between them. Recently, Muehsam and Pilla [63, 64] proposed a Lorenz model for weak magnetic field bioeffects, suggesting that weak exogenous AC/DC magnetic fields can act on an ion/ligand bound in a molecular cleft, based upon the assumption that the receptor molecule is able to detect the Larmor trajectory of an ion or ligand within the binding site. To date, however, there is insufficient direct experimental evidence pertaining to this model. Further studies are required to better understand the mechanisms of SMF bioeffects, in particular, for the interaction between bone hemodynamics and bone mineralization.

## 5. Conclusion

Exposure to the SMF at *B*_max_ of 180 mT and *G*_max_ of 110 mT mm^−1^ significantly increased the BMD values of the osteoporotic lumbar vertebrae in ovariectomized rats without significantly influencing the E_2_ levels. It seemed possible that the improvement of BMD values caused by SMF could be partially due to increased circulation in bone and bone marrow.
